# TBK1 adaptor AZI2/NAP1 regulates NDP52-driven mitochondrial autophagy

**DOI:** 10.1016/j.jbc.2024.107775

**Published:** 2024-09-12

**Authors:** Ryu Endo, Hiroki Kinefuchi, Momoha Sawada, Reika Kikuchi, Waka Kojima, Noriyuki Matsuda, Koji Yamano

**Affiliations:** Department of Biomolecular Pathogenesis, Medical Research Institute, Tokyo Medical and Dental University, Bunkyo-ku, Tokyo, Japan

**Keywords:** autophagy, mitochondria, mitophagy, polyubiquitin chain, serine/threonine protein kinase

## Abstract

Damaged mitochondria are selectively eliminated in a process called mitophagy. PINK1 and Parkin amplify ubiquitin signals on damaged mitochondria, which are then recognized by autophagy adaptors to induce local autophagosome formation. NDP52 and OPTN, two essential mitophagy adaptors, facilitate *de novo* synthesis of pre-autophagosomal membranes near damaged mitochondria by linking ubiquitinated mitochondria and ATG8 family proteins and by recruiting core autophagy initiation components. The multifunctional serine/threonine kinase TBK1 also plays an important role in mitophagy. OPTN directly binds TBK1 to form a positive feedback loop for isolation membrane expansion. TBK1 is also thought to indirectly interact with NDP52; however, its role in NDP52-driven mitophagy remains largely unknown. Here, we focused on two TBK1 adaptors, AZI2/NAP1 and TBKBP1/SINTBAD, that are thought to mediate the TBK1-NDP52 interaction. We found that both AZI2 and TBKBP1 are recruited to damaged mitochondria during Parkin-mediated mitophagy. Further, a series of *AZI2* and *TBKBP1* knockout constructs combined with an *OPTN* knockout showed that AZI2, but not TBKBP1, impacts NDP52-driven mitophagy. In addition, we found that AZI2 at S318 is phosphorylated during mitophagy, the impairment of which slightly inhibits mitochondrial degradation. These results suggest that AZI2, in concert with TBK1, plays an important role in NDP52-driven mitophagy.

TANK-binding kinase 1 (TBK1) is a multifunctional serine/threonine kinase that impacts a broad range of intracellular functions including innate immunity, cell cycle, and autophagy (1). TBK1 function, however, is influenced by the adaptors to which it binds. TBK1 consists of an N-terminal kinase domain (KD) followed by a ubiquitin-like domain, a helical scaffold dimerization domain (SDD) and a C-terminal domain (2). For example, in the cGAS-STING signaling pathway, the C-terminal STING peptide interacts with TBK1 *via* a groove formed by the KD and SDD (3, 4). The C-terminal domain of TBK1 also functions as a scaffold to bind different proteins including TANK, OPTN/Optineurin, AZI2/NAP1, and TBKBP1/SINTBAD that regulate IRF-dependent transcription and autophagy (5).

Macroautophagy (hereafter referred to as autophagy) is a lysosome-dependent intracellular degradation process that maintains cellular homeostasis *via* degradation of harmful and/or unwanted cytosolic compartments and organelles (6). In contrast to bulk non-selective autophagy, ubiquitin-dependent selective autophagy relies on autophagy adaptors (also known as autophagy receptors) to bridge ubiquitinated cargo and the autophagy machinery (7, 8). Currently, five autophagy adaptors have been identified in mammals with NDP52 and OPTN essential for Parkin-mediated mitophagy and TAX1BP1 only marginally important (9). Following a loss in the membrane potential, damaged mitochondria are ubiquitylated through the concerted actions of the ubiquitin kinase PINK1 and the ubiquitin ligase Parkin (10, 11, 12, 13, 14). OPTN is then recruited to the contact site between the damaged mitochondria and the autophagosome formation site. The N-terminal coiled-coil of OPTN is required for direct interaction with TBK1 (15), whereas the leucine zipper and LIR motifs are responsible for interactions with the ATG9A vesicle (16) and ATG8 family proteins (17). TBK1 activation at the autophagosome formation site triggers its phosphorylation of OPTN S177 near the LIR motif (17), which in turn induces interactions with FIP200, the upstream autophagy initiation subunit (18). Furthermore, OPTN-TBK1 forms a positive feedback loop that amplifies the phosphorylation signal on the autophagosome formation site to induce *de novo* synthesis of the isolation membrane (19, 20).

Another essential mitophagy adaptor, NDP52, also interacts with TBK1 and a subunit of the autophagy initiation complex. NDP52 contains an N-terminal SKIP carboxyl homology (SKICH) domain, a long coiled-coil domain responsible for NDP52 dimerization, and two C-terminal zinc-finger domains for binding ubiquitin chains. While NDP52 also has an LIR that facilitates associations with LC3-labelled isolation membranes (21), the SKICH domain directly interacts with FIP200 (22, 23, 24, 25), which recruits the autophagy initiation complex to damaged mitochondria. The NDP52 SKICH domain also mediates interactions with the TBK1-binding adaptors AZI2 and TBKBP1. Both AZI2 and TBKBP1 have similar domain architecture including several coiled-coil domains with NDP52 interactions localized to the N-terminus and TBK1 interactions to the C-terminus (15, 26). AZI2 and TBKBP1 are assumed to recruit TBK1 to a region adjacent to the autophagosome formation site. While AZI2 and TBKBP1 are important for xenophagy (24, 27) and TBK1-mediated immune responses (28, 29, 30), their specific functions in mitophagy remain to be elucidated. The kinase activity of TBK1 is important for PINK1-Parkin-mediated mitophagy, but it is unclear how it impacts OPTN and NDP52. Our previous study of the OPTN-TBK1 axis revealed that TBK1 initiates isolation membrane formation near damaged mitochondria (20), but it is unclear if this is the case for NDP52-driven mitophagy.

In this study, we made and used a series of individual and combinatorial knockout cell lines for *AZI2*, *TBKBP1*, and *OPTN*, to determine that AZI2, but not TBKBP1, is required for NDP52-driven mitophagy.

## Results

### Subcellular localization of the TBK1 adaptors AZI2 and TBKBP1 during mitophagy

**Figure 1:**
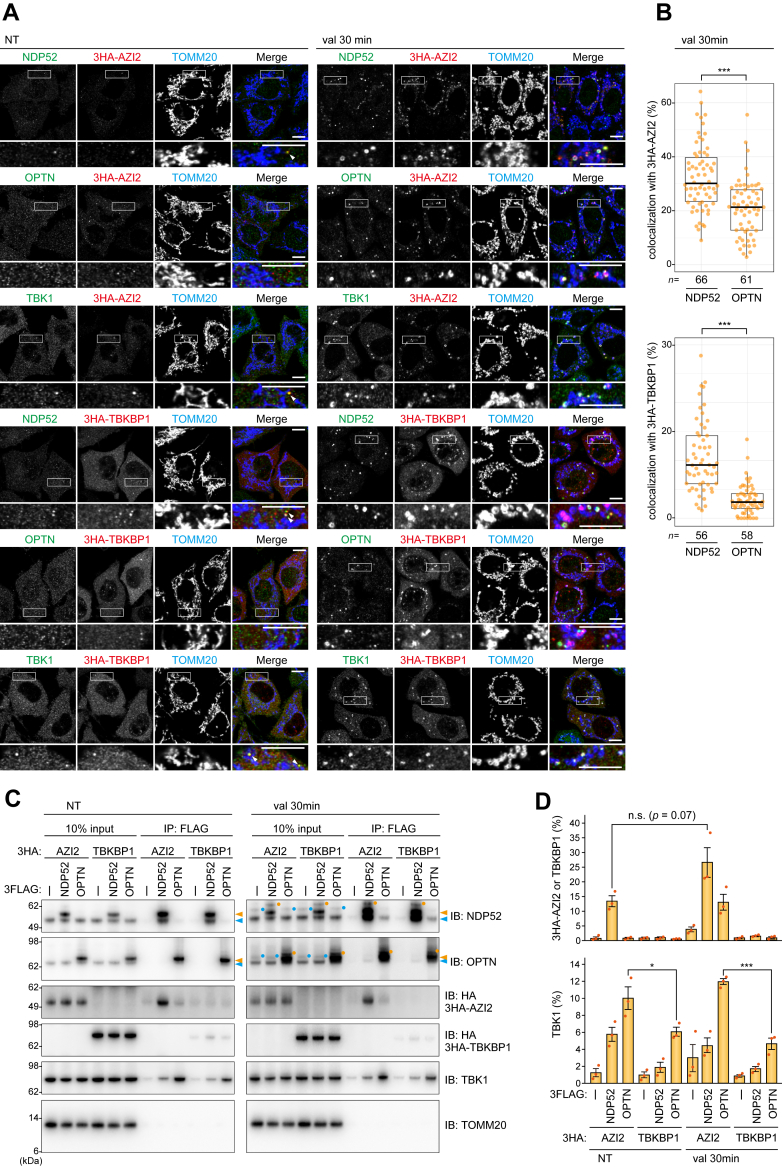
**Subcellular localization of TBK1 adaptors upon mitophagy**. *A*, parkin-AAVS-HeLa cells stably expressing 3HA-AZI2 and 3HA-TBKBP1 were treated without (NT, non-treated) or with valinomycin (val 30 min). The cells were immunostained with the indicated antibodies. Magnified images of the regions bounded by the *white* rectangles are shown. The *white* arrowheads indicate cytosolic AZI2/TBKBP1 dots colocalizing with NDP52 or TBK1. All images are representative of at least two independent experiments. Bars, 10 μm. *B*, the colocalization of 3HA-AZI2 or 3HA-TBKBP1 with endogenous NDP52 and OPTN after 30 min of valinomycin treatment in (*A*) was quantified. The data are shown as a box plot, with the box indicating the interquartile range (IQR), the whiskers corresponding to the range of values that are within 1.5 × IQR, and the horizontal line indicating the median. More than 56 cells (*n*) for each treatment were examined across two independent experiments. *C*, Parkin-AAVS-HeLa cells stably expressing the indicated 3FLAG-tagged and 3HA-tagged proteins were treated without (NT) or with valinomycin (val 30 min). The cells were treated with a DSP crosslinker and FLAG-tagged proteins were immunoprecipitated using an anti-FLAG antibody. Light *blue* and *orange* arrowheads indicate endogenous and 3FLAG-tagged proteins, respectively. Light *blue* and *orange* dots indicate ubiquitinated bands. All blots are representative of at least two independent experiments. *D*, Co-immunoprecipitants from the 3FLAG-NDP52 or 3FLAG-OPTN pull-downs in (*C*) were quantified. Error bars represent mean ± s.e. of three independent experiments.

Because OPTN and NDP52 are recruited to damaged mitochondria following a loss in the mitochondrial membrane potential (31), we initially examined the subcellular localization of AZI2 and TBKBP1 during mitophagy. 3HA-tagged AZI2 (3HA-AZI2) and 3HA-tagged TBKBP1 (3HA-TBKBP1) were stably expressed in Parkin-expressing HeLa cells, and the membrane potential was dissipated by the addition of valinomycin to induce mitophagy. Under normal growth conditions, NDP52, OPTN, TBK1, 3HA-AZI2, and 3HA-TBKBP1 are localized in the cytosol (Fig. 1*A*). As expected, OPTN and NDP52 were recruited to mitochondria after 30 min valinomycin treatment (Fig .1*A*). 3HA-AZI2 and 3HA-TBKBP1 were also recruited to mitochondria where they colocalized to a high degree with NDP52 (Fig. 1, *A* and *B*). In contrast, their colocalization with OPTN was sparse (Fig. 1, *A* and *B*). Furthermore, endogenous TBK1 was found on damaged mitochondria and was almost completely colocalized with 3HA-AZI2 and 3HA-TBKBP1 (Fig. 1*A*). These results indicate that AZI2 and TBKBP1 are recruited to regions near damaged mitochondria where NDP52 and TBK1 are assembled. In addition, 3HA-AZI2 and 3HA-TBKBP1 form small dot-like structures that colocalize with cytosolic NDP52 and TBK1 even under non-mitophagic conditions (Fig. 1*A*, indicated by white arrowheads). To examine the nature of these interactions, co-immunoprecipitation assays were done using cells expressing 3FLAG-tagged NDP52 (3FLAG-NDP52) or OPTN (3FLAG-OPTN) along with either 3HA-AZI2 or 3HA-TBKBP1. The cells were treated with or without valinomycin for 30 min and then DSP-crosslinked. Immunoprecipitation of 3FLAG-NDP52 yielded 3HA-AZI2 and endogenous TBK1 in the bound fractions (Fig. 1*C*). While 3FLAG-OPTN pulled down TBK1, it did not interact with either AZI2 or TBKBP1 (Fig. 1*C*). Furthermore, when compared to the negative control (none), a small amount of TBKBP1 was co-immunoprecipitated with 3FLAG-NDP52 (Fig. 1*C*). Interestingly, while valinomycin induced ubiquitination of NDP52 and OPTN (20), there was only a slight increase in AZI2 co-immunoprecipitated with 3FLAG-NDP52 relative to the non-treated samples (Fig. 1, *C* and *D*). No evidence was found for TBKBP1-NDP52 interactions even under mitophagic conditions (Fig. 1, *C* and *D*). These results suggest that NDP52 constitutively interacts with TBK1 and AZI2. Furthermore, quantitative analysis of TBK1 co-immunoprecipitated with 3FLAG-OPTN revealed that their interaction was partially inhibited by TBKBP1 overexpression (Fig. 1*D*), suggesting that OPTN and TBKBP1 compete for TBK1.

### AZI2 is crucial for NDP52-driven mitophagy

**Figure 2:**
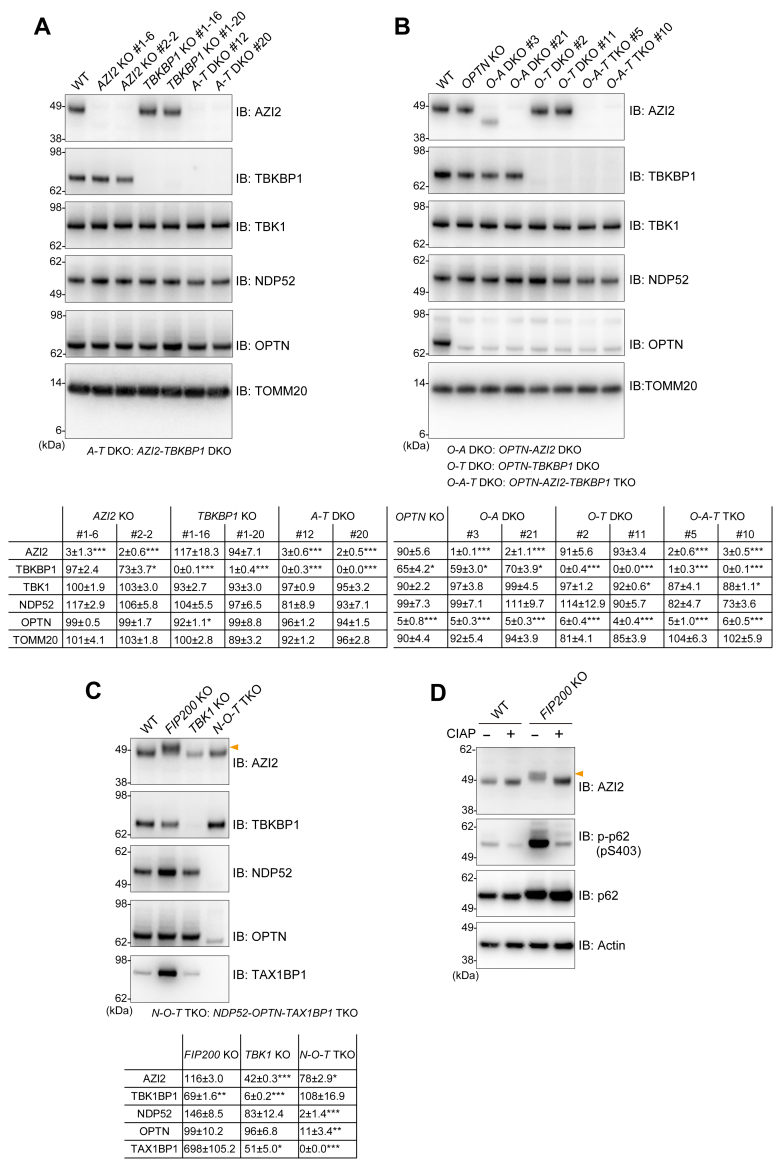
**AZI2 and TBKBP1 gene knockout in HeLa cells**. *A*–*C*, total cell lysates prepared from the indicated cell lines were analyzed by immunoblotting with the indicated antibodies. The *orange* arrowhead indicates an upward AZI2 band shift in *FIP200* KO cells. Protein levels (mean ± s.e.) in the indicated cell lines are shown in tables below each blot. Protein levels were normalized to that of WT cells (100%). Values were calculated from three independent experiments. *D*, cell lysates extracted from WT and *FIP200* KO cells were incubated with recombinant phosphatase (CIAP) and immunoblotted. The *orange* arrowhead indicates a phosphorylated form of AZI2.

To investigate the roles of AZI2 and TBKBP1 in Parkin-mediated mitophagy, *AZI2* and/or *TBKBP1* were knocked out in HeLa cells using CRISPR/Cas9 editing (Fig. 2*A*). Since NDP52 and OPTN function redundantly in mitophagy, we also knocked out these genes in an *OPTN* KO HeLa line (Fig. 2*B*). KO of the target genes was confirmed by immunoblotting (Fig. 2, *A* and *B*) as well as genome sequencing (Table. S1). Immunoblotting showed that depletion of AZI2 and TBKBP1 either singly or in tandem did not significantly impact the stability of NDP52, OPTN, or TBK1 (Fig. 2*A*). *OPTN* deletion, however, slightly reduced TBKBP1 levels (Fig. 2*B*). Although NDP52 levels in our *OPTN-AZI2-TBKBP1* TKO HeLa cells were also slightly reduced, they were not significantly different from WT (Fig. 2*B*). TBK1 levels in one of the TKO clones was similarly impacted, albeit with marginal significance relative to WT. This reduction though was limited to the one clone (Fig. 2*B*). Next, we examined whether the loss of the selective autophagy components, including TBK1, affected the stability of AZI2 and TBKBP1. FIP200, an essential subunit of the autophagy initiation complex that is thought to directly recruit autophagy adaptors to autophagosome formation sites, was recently shown to bind the N-terminal region of AZI2 (25). As shown in Fig. 2*C*, depletion of FIP200 caused TBKBP1 instability and affected the electrophoretic migration of AZI2 (Fig. 2*C*). Given that *TBK1* deletion restores the non-modified migration pattern (20), the slower migrating AZI2 band likely corresponds to a TBK1-mediated phosphorylated form. *In vitro* phosphatase treatment likewise restored the electrophoretic mobility of AZI2 (Fig. 2*D*). The basal autophagy inhibition that results from *FIP200* deletion drives AZI2 accumulation within cytosolic condensates facilitating AZI2 phosphorylation by activated TBK1. While the protein levels of AZI2 and TBKBP1 were not severely affected by depletion of the NDP52, OPTN, and TAX1BP1 autophagy adaptors, AZI2 in *TBK1* KO cells was significantly reduced relative to WT cells and TBKBP1 was barely detectable (Fig. 2*C*). Our results indicate that the protein stability and/or post-translational status of AZI2 and TBKBP1 is affected by the macroautophagy machinery and TBK1.

**Figure 3:**
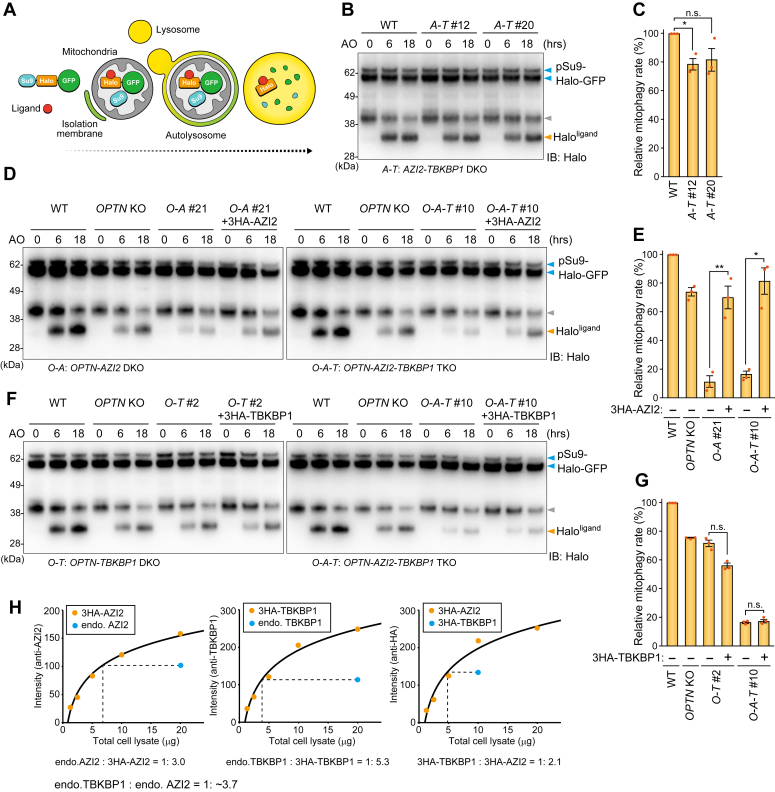
**TBK1 adaptors AZI2 and TBKBP1 impact NDP52-driven mitophagy**. *A*, schematic representation of the mitophagy Halo Tag processing assay. *B, D, and F*, cells stably expressing pSu9-Halo-mGFP and Parkin were treated with Halo^ligand^. After pulse-labeling, the cells were treated with antimycin A and oligomycin (AO) for the indicated times and analyzed by immunoblotting. The *gray* arrowheads indicate ∼40 kDa bands that are likely a cleavage product of the HaloTag construct generated during GFP chromophore formation. *C, E, and G*, The mitophagy flux in (B, D, F) was quantified. The ratio of Halo^ligand^ to pSu9-Halo-mGFP after AO 18 h was calculated. The WT score was set to 100. Error bars represent mean ± s.e. of three independent experiments. *H*, comparison of endogenous AZI2 and TBKBP1 protein levels and the exogenous 3HA-tags.

Next, we investigated whether *AZI2* and/or *TBKBP1* deletions impact Parkin-mediated mitophagy. For this purpose, we used a HaloTag processing assay to monitor the mitophagy flux (32). pSu9-Halo-GFP and Parkin were stably expressed in HeLa cells and mitophagy was induced by the addition of antimycin A and oligomycin (AO) in the presence of a HaloTag ligand (Fig. 3*A*). AO was used to detect lysosomal degradation of mitochondrial Halo-GFP rather than valinomycin because its extended use neutralizes lysosomes and impairs efficient mitochondrial degradation (9, 16). As expected, a ∼33 kDa Halo^ligand^ band appeared in a time-dependent manner in WT cells following AO treatment, indicating mitochondrial degradation (Fig. 3*B*). In *AZI2-TBKBP1* DKO cells, a minor reduction in the mitophagy flux was observed with a marginally significant difference relative to WT cells observed in clone #12 but not clone #20 (Fig. 3, *B* and *C*). Since OPTN drives mitophagy independently of NDP52, we also investigated the mitophagy flux in *OPTN*-deleted cells. Although mitophagy is slightly reduced by *OPTN* KO, Halo^ligand^ bands were barely detectable in *OPTN-AZI2* DKO and *OPTN-AZI2-TBKBP1* TKO cells (Fig. 3*D*). Importantly, the expression of exogenous 3HA-AZI2 in these cells recovered the mitophagy flux (Fig. 3, *D* and *E*), indicating that AZI2 is crucial for NDP52-driven mitophagy. In contrast, deletion of *TBKBP1* in *OPTN* KO cells (*OPTN-TBKBP1* DKO cells) did not inhibit the mitophagy flux (Fig. 3, *F* and *G*). Since the amount of endogenous AZI2 protein was ∼3.7 times higher than that of endogenous TBKBP1 in our HeLa cells (Fig. 3*H*), we initially assumed that it was the reason for the strong inhibitory effect of the AZI2 deletion on mitophagy. However, overexpression of 3HA-TBKBP1, which surpassed endogenous AZI2 protein levels, was unable to recover the mitophagy defect in *OPTN-AZI2-TBKBP1* TKO cells (Fig. 3, *E* and *F*). We thus concluded that AZI2, but not TBKBP1, is important for NDP52-driven mitophagy.

### Deletion of the TBK1 adaptor AZI2 impairs an early step in isolation membrane formation

**Figure 4:**
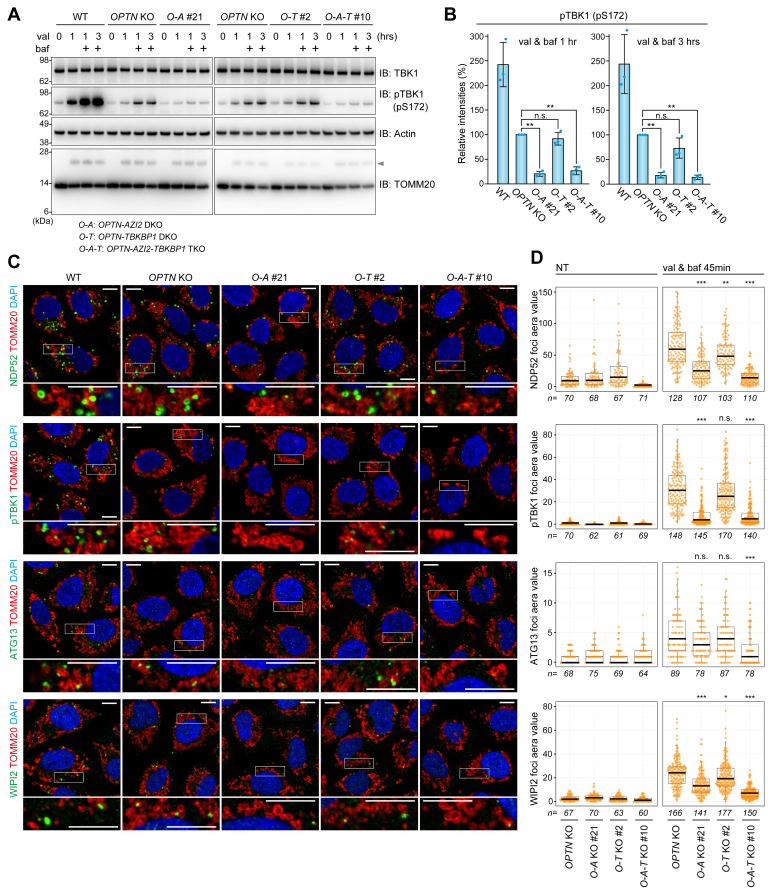
**Recruitment of the NDP52 and autophagy core subunits to damaged mitochondria is inhibited by the loss of TBK1 adaptors**. *A*, cells stably expressing Parkin were treated with valinomycin (val) in the presence or absence of bafilomycin A1 (baf). Total cell lysates were analyzed by immunoblotting. The *gray* arrowhead indicates ubiquitinated TOMM20. All blots are representative of at least two independent experiments. *B*, the levels of phosphorylated TBK1 at S172 in (*A*) were quantified and normalized to levels in *OPTN* KO cells, which were set to 100. Error bars represent mean ± s.e. of three independent experiments. *C*, cells stably expressing Parkin were treated with val and baf for 45 min and immunostained with the indicated antibodies. Magnified images of the regions bounded by the *white* rectangles are shown. Bars, 10 μm. *D*, foci formation of the indicated proteins on mitochondria after cells were treated without (NT) or with val and baf for 45 min in (*C*) were quantified. The data are shown as a box plot, with the box indicating the interquartile range (IQR), the whiskers correspond to the range of values that are within 1.5 × IQR, and the horizontal line indicates the median. More than 60 cells (*n*) from each cell line were examined in two independent experiments.

Which steps in mitophagy are inhibited by the deletion of the TBK1 adaptors? To address this question, we first detected phosphorylated TBK1 (pTBK1) during mitophagy. We previously showed that pTBK1 levels correlate with autophagosome formation efficiency during OPTN-driven mitophagy (20). In response to mitophagy induction, TBK1 is activated *via* autophosphorylation of S172 (33), followed immediately by autophagy-dependent lysosomal degradation. Consequently, to accurately detect phosphorylated TBK1, we also treated cells with bafilomycin A1 (an inhibitor of autophagosome-lysosome fusion). Substantial TBK1 phosphorylation was observed in WT cells (Fig. 4*A*). While pTBK1 levels were reduced following *OPTN* KO relative to WT, a time-dependent increase was still evident (Fig. 4*A*). A mitophagy-dependent increase in pTBK1, however, was barely detectable in *OPTN-AZI2* DKO and *OPTN-AZI2-TBKBP1* TKO cells (Fig. 4, *A* and *B*). This result is consistent with the HaloTag data and strongly suggests that the defective mitophagy flux is due to impaired TBK1 activation. Next, we observed NDP52 recruitment to damaged mitochondria in the KO cell lines. After mitophagy induction, NDP52 in both WT and *OPTN* KO cells formed cup-shaped and/or sphere-like structures on mitochondria (Fig. 4*C*) that were valinomycin dependent (Fig. 4*D*). The abundance of these structures was reduced in the *OPTN-AZI2* DKO and *OPTN-AZI2-TBKBP1* TKO cells (Fig. 4, *C* and *D*). Furthermore, pTBK1 accumulation was reduced in *OPTN* KO cells by further deletion of *AZI2* (Fig. 4, *C* and *D*), which is consistent with the immunoblotting results (Fig. 4, *A* and *B*). Since NDP52 interacts with an autophagy initiation complex that includes FIP200 and ATG13 (22, 23, 24, 25), we sought to observe endogenous ATG13 recruitment to damaged mitochondria. Although the number of mitophagic ATG13 foci was lower than that of NDP52, ATG13 foci formation in *OPTN* KO cells was evident (Fig. 4*C*). In sharp contrast, these structures were largely absent in *OPTN-AZI2* DKO and *OPTN-AZI2-TBKBP1* TKO cells (Fig. 4, *C* and *D*). Similar results were obtained when we observed the recruitment of endogenous WIPI2 (Fig. 4, *C* and *D*). We further confirmed that the generation of the pTBK1 signal and the recruitment of ATG13 and WIPI2 to mitochondria were absent in all cell lines under non-mitophagic conditions (Fig. 4*D*). Taken together, these results indicate that AZI2 depletion inhibits mitophagy-dependent TBK1 activation and the recruitment of NDP52 and ATG13 (*i.e.*, the initial components required for isolation membrane formation). As a result, later steps in autophagosome formation, such as WIPI2 recruitment, would also be impaired.

### AZI2 S318 phosphorylation is important for NDP52-driven mitophagy

**Figure 5:**
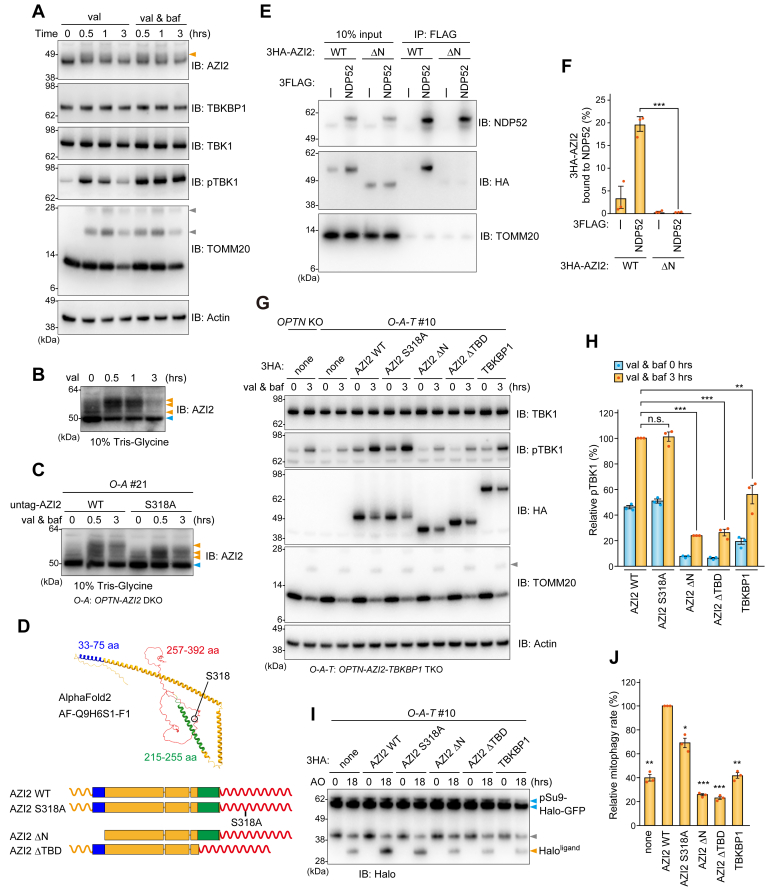
**Identification of AZI2 domains required for NDP52-driven mitophagy**. *A*, AAVS-Parkin HeLa cells were treated with valinomycin (val) and bafilomycin A1 (baf) for the indicated times and total cell lysates were analyzed by immunoblotting. The *orange* and *gray* arrowheads indicate slower migrating AZI2 and ubiquitinated TOMM20 bands, respectively. All blots are representative of at least two independent experiments. *B*, the samples prepared in (*A*) were analyzed by 10% Tris-Glycine SDS-PAGE followed by immunoblotting. Light *blue* and *orange* arrowheads indicate unmodified and phosphorylated AZI2, respectively. All blots are representative of at least two independent experiments. *C*, *OPTN-AZI2* DKO HeLa cells stably expressing Parkin and untagged AZI2 WT or S318 A were analyzed as in (*B*). All blots are representative of at least two independent experiments. *D*, a predicted AZI2 structure (AlphaFold2 AF-Q9H6S1-F1) is shown. The N-terminal region corresponding to amino acids 33 to 75 that interact with NDP52, amino acids 215 to 255 that interact with TBK1, and the C-terminal unstructured region are colored *blue*, *green*, and *red*, respectively. A schematic diagram of the AZI2 mutants used in this study is shown below. *E*, *OPTN-AZI2*-*TBKBP1* TKO HeLa cells stably expressing Parkin, 3FLAG-NDP52, 3HA-AZI2 WT, or a ΔN mutant were treated with a DSP crosslinker and 3FLAG-NDP52 was immunoprecipitated with an anti-FLAG antibody. All blots are representative of at least two independent experiments. *F*, Co-immunoprecipitated AZI2 in (*E*) was quantified. Error bars represent mean ± s.e. of three independent experiments. *G*, cells stably expressing Parkin were treated with val and baf for 3 h. The total cell lysates were analyzed by immunoblotting. The *gray* arrowhead indicates ubiquitinated TOMM20. All blots are representative of at least two independent experiments. *H*, the levels of phosphorylated TBK1 at S172 (pTBK1) in (G) were quantified. The amount of pTBK1 in *OPTN-AZI2-TBKBP1* TKO cells with overexpression of AZI2 WT after val and baf treatment was set to 100. Error bars represent mean ± s.e. of three independent experiments. *I*, cells stably expressing pSu9-Halo-mGFP and Parkin were used with the Halo Tag processing assay. After pulse-labelling, the cells were treated with antimycin A and oligomycin (AO) for 18 h and analyzed by immunoblotting. The *gray* arrowheads indicate ∼40 kDa bands that are likely a cleavage product of the HaloTag construct generated during GFP chromophore formation. All blots are representative of at least two independent experiments. *J*, the mitophagy flux in (*I*) was quantified. The ratio of Halo^ligand^ to pSu9-Halo-mGFP after 18 h AO was calculated. The score in *OPTN-AZI2-TBKBP1* TKO cells with overexpression of AZI2 WT was set to 100. Error bars represent mean ± s.e. of three independent experiments.

In the mitophagy assay, we noticed an expansion of the AZI2 immunoreactive band on 4 to 12% Bis-Tris NuPAGE gels in cells post-mitophagy induction (Fig. 5*A*). Differences in AZI2 mobility were more clearly observed when a conventional Tris-Glycine gel was used (Fig. 5*B*). This shift in electrophoretic mobility is suggestive of TBK1-dependent phosphorylation. We previously showed that TBK1 phosphorylates AZI2 when basal autophagy is inhibited, and Paul *et al.* recently showed that AZI2 is phosphorylated at S318 by TBK1 in response to mitosis progression (20, 34). Correspondingly, the slowest migrating AZI2 band was abolished by an S318 A mutation (Fig. 5*C*), indicating that AZI2 S318 is phosphorylated in response to Parkin-mediated mitophagy. AlphaFold2 predicts that AZI2 consists of an N-terminal long coiled-coil region and a C-terminal intrinsically disordered region (Fig. 5*D*). NDP52 and TBK1 have been reported to bind to amino acids 33 to 75 (26) and 215 to 255 (15) in AZI2, respectively. Using co-immunoprecipitation assays, we confirmed that the interaction between AZI2 and NDP52 requires the N-terminal 75 amino acids of AZI2 (Fig. 5, *E* and *F*). We next sought to identify the AZI2 domain responsible for NDP52-driven mitophagy. Expression of WT AZI2 in *OPTN-AZI2-TBKBP1* TKO cells resulted in considerable pTBK1 accumulation under non-mitophagic conditions which increased further following mitophagy induction (Fig. 5, *G* and *H*). The increase in pTBK1 levels following Parkin-mediated mitophagy was inhibited by deletions to the N-terminal domain as well as the TBK1-binding domain (TBD) (Fig. 5, *G* and *H*). However, the S318 A mutation did not inhibit mitophagy-dependent pTBK1 production (Fig. 5, *G* and *H*). The HaloTag mitophagy assay showed that AZI2 mutants lacking either the N-terminal domain or TBD were unable to fully proceed to mitophagy and that mitochondrial degradation was marginally inhibited by the S318 A mutation (Fig. 5, *I* and *J*). These findings indicate that, in addition to the TBD, the NDP52-interacting N-terminal region of AZI2 is also required for mitophagy progression. Although the effect was weak, phosphorylation of AZI2 at S318 may be important for efficient mitophagy. Overexpression of TBKBP1 in *OPTN-AZI2-TBKBP1* TKO cells increased mitophagy-dependent pTBK1 production (Fig. 5, *G* and *H*), but it could not recover the mitophagy defect (Fig. 5, *I* and *J*).

## Discussion

OPTN, AZI2, and TBKBP1 are thought to be adaptors that directly interact with TBK1 to regulate its activation. Previously, we and others revealed that OPTN is one of the essential adaptors and that OPTN-mediated activation of TBK1 following recruitment to damaged mitochondria regulates autophagosome formation through a positive feedback loop (19, 20, 33). This model is supported by the finding that TBK1 impairment inhibits Parkin-mediated mitophagy (35). To clarify the functions of other TBK1 adaptors in mitophagy, we initially generated cell lines with *AZI2* and *TBK1BP1* knocked out either singly or in tandem, but these deletions had minimal inhibitory effects on mitochondrial degradation (Fig. 3, *B* and *C*).

Since OPTN and NDP52 independently and redundantly function as mitophagy adaptors (19), we next knocked out *AZI2* and *TBKBP1* in *OPTN* KO cells to establish mitophagy conditions dependent only on NDP52 and examined the respective functions of AZI2 and TBKBP1 in Parkin-mediated mitophagy. Although AZI2 and TBKBP1 have similar domain structures, we found that only AZI2 was required for NDP52-driven mitophagy. Knockout of *AZI2* impaired NDP52 recruitment to damaged mitochondria, TBK1 activation on damaged mitochondria (Fig. 4), and mitochondrial degradation (Fig. 3, *D* and *E*). Like OPTN-mediated mitophagy, our results strongly suggest that AZI2 mediates the interaction between NDP52 and TBK1 and activates TBK1 *via* its accumulation at points of contact between damaged mitochondria and the autophagosome formation site. Nguyen *et al.* suggested that in addition to regulating mitophagy through phosphorylation, TBK1 also acts as a scaffold for autophagosome formation by interacting with the phosphatidylinositol 3-kinase complex (19). Consequently, AZI2 may drive mitophagy *via* TBK1 recruitment to damaged mitochondria.

While activated TBK1 on damaged mitochondria phosphorylated AZI2 including S318, the effect of the S318 A phospho-deficient mutant on NDP52-driven mitophagy was minimal (Fig. 5, *I* and *J*). Furthermore, the presence of multiple slower migrating AZI2 bands under mitophagic conditions despite the S318 A mutation (Fig. 5*C*), suggests that additional phosphorylation sites exist in AZI2. During manuscript preparation, Adriaenssens *et al.* reported that several serine residues (S34, S82, S83, S120, S318, and S343) in AZI2 are phosphorylated in response to mitophagy (36). How the intramolecular conformation of AZI2 and/or its intermolecular interactions are impacted by the phosphorylation events and how those events affect mitophagy remain to be investigated. Even though the interaction between OPTN and TBK1 was slightly inhibited by TBKBP1 overexpression (Fig. 1*D*), which is consistent with the recent report by Adriaenssens *et al.* (36), *TBKBP1* KO did not affect NDP52-driven mitophagy (Fig. 3, *F* and *G*). Even though TBKBP1 interacts with both NDP52 and TAX1BP1, its role in TAX1BP1-mediated selective autophagies such as aggrephagy (37, 38) and ferritinophagy (39) remains to be explored. Similarly, the *in vivo* function of AZI2 has yet to be fully clarified. Although *AZI2* knockout mice develop severe osteoporosis due to enhanced osteoclast survival (40), no mitophagy-related phenotypes associated with AZI2 deficiency have been reported *in vivo*.

**Figure 6:**
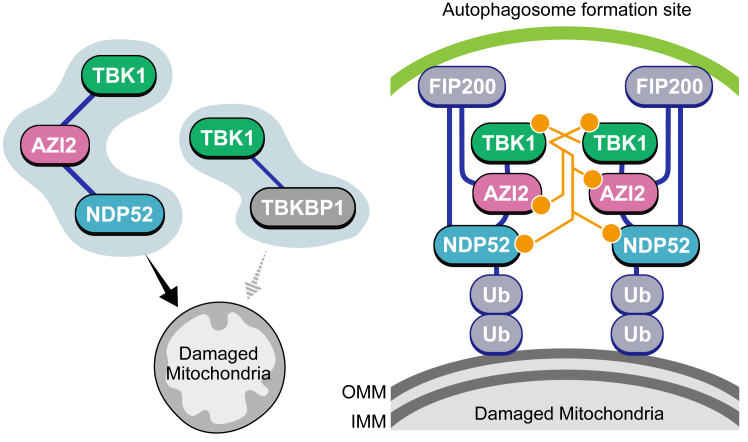
**Proposed model of NDP52-dependent mitophagy initiation. Under basal conditions, AZI2-TBK1, but not TBKBP1-TBK1 interacts with NDP52**. The dark blue lines connecting each protein denote protein-protein interactions. Upon mitophagy stimulation, the NDP52-AZI2-TBK1 complex is recruited to ubiquitin (Ub)-coated damaged mitochondria where the multiple complexes are assembled at the autophagosome formation site through interactions such as between NDP52 and FIP200. The proximity of TBK1 to each other induces TBK1 hetero-autophosphorylation. AZI2 and NDP52 are then phosphorylated by the activated TBK1, which expand an isolation membrane. *Orange* dots denote phosphorylation sites. OMM, outer mitochondrial membrane; IMM, inner mitochondrial membrane.

In summary, our study revealed a new additional layer for TBK1 activation upon Parkin-mediated mitophagy (Fig. 6). Although OPTN functions as a dominant adaptor for TBK1 activation, AZI2 also plays an important role in TBK1 activation in an NDP52-dependent manner. AZI2 interacts with both TBK1 and NDP52 under basal conditions, and the AZI2 complex is recruited to ubiquitin-coated damaged mitochondria where the multiple AZI2 complexes are assembled at the autophagosome formation site through various interaction axes such as NDP52-FIP200, and AZI2-FIP200. Since TBK1 hetero-autophosphorylation is important for mitophagy initiation, AZI2 will enable damaged mitochondria to be eliminated efficiently in various cellular demands and/or pathological conditions.

## Experimental procedures

### Materials and methods

Reagents including cell lines, antibodies, and plasmid DNAs used in this study are listed in Tables S2–S4, respectively.

### Cell culture and transfection

HeLa and HEK293T cells were cultured in DMEM supplemented with 10% FBS, 1 mM sodium pyruvate, nonessential amino acids, and PSG. *OPTN* KO HeLa (9), *NDP52/OPTN/TAX1BP1* TKO HeLa (9), *FIP200* KO HeLa (23), *TBK1* KO HeLa (20), and AAVS-Parkin HeLa (20) cell lines were described previously. Stable cell lines were made by recombinant retrovirus infection (16). Cell lines used in this study were authenticated and tested for *mycoplasma* contamination. FuGENE six and FuGENE HD reagents were used for plasmid transfection according to the manufacturer’s instructions.

### Gene knockout using CRISPR/Cas9 gene editing

gRNA target sequences for AZI2 exon three and TBKBP1 exon three were designed using CRISPOR (http://crispor.tefor.net/). Each of the oligonucleotide pairs was annealed and introduced into the BpiI site of the PX459 vector. The resultant plasmids were transfected into HeLa cell lines. Puromycin-resistant cells were seeded into 96-well plates, and single clones were analyzed by immunoblotting and genome sequencing to confirm target gene knockout.

### Immunoblotting

The preparation of total cell lysates was described previously (20). Total cell lysates were loaded on NuPAGE 4 to 12% Bis-Tris gels or SDS-PAGE 10% Tris-Glycine gels. Proteins were transferred to PVDF membranes that were blocked with 2% (w/v) skim-milk/TBS-T and then incubated with primary and HRP-conjugated secondary antibodies. Proteins were detected using a Western Lighting Plus-ECL Kit on a FUSION SOLO S system (VILBER). ImageJ was used to quantify protein bands.

### Immunostaining

The preparation of immunostained cells was described previously (20). Microscopy images were captured using inverted LSM710 and LSM780 confocal microscopes (CarlZeiss) with a Plan-Apochromat 63x/1.4 oil DIC lens or an FV3000 (EVIDENT) confocal microscope with a PlanApo N 60x/1.4 oil objective lens. Photoshop (Adobe) and ImageJ software were used for image analysis. Quantification of endogenous NDP52, pTBK1, ATG13, and WIPI2 foci formation after mitophagy induction was performed as follows. Seven microscopy image slices with 1 μm z-stack intervals were combined using the maximum intensity projection. The signals in the nuclei were subtracted based on DAPI staining since non-specific nuclear signals can occur with the WIPI2 and pTBK1 antibodies. These area values colocalized with mitochondria (immunostained with anti-TOMM20 antibody) in each cell above an appropriate preset threshold were calculated using ImageJ software. More than 60 cells from two independent experiments were quantified.

### Mitophagy assay using pSu9-Halo-mGFP

Cells stably expressing Parkin and pSu9-Halo-mGFP were pre-treated with 100 nM TMR-conjugated Halo ligand for 20 min. After washing twice with PBS, the cells were cultivated in DMEM with 10 μM oligomycin, 4 μM antimycin A, and 10 μM Q-VD to induce mitophagy. Total cell lysates were subsequently prepared.

### Co-immunoprecipitation

Cells grown in 6-well plates were washed twice with PBS and incubated with 1 mM DSP (dithiobis(succinimidyl propionate)) in PBS at room temperature for 30 min. The crosslink reaction was quenched by the addition of 400 mM Tris-HCl pH7.5. After a TBS buffer (50 mM Tris-HCl pH7.5, 120 mM NaCl) wash, the cells were solubilized with 600 μl of 1% TX-100 buffer (50 mM Tris-HCl pH7.5, 150 mM NaCl, 1%[v/v] Triton X-100, protease inhibitor cocktail, 10 μM MLN7243) on ice for 10 min. Insolubilized debris was removed by centrifugation at 14,000×*g* for 5 min at 4 °C and 500 μl of the supernatants were mixed with 20 μl of equilibrated 50% (v/v) DDDDK-tagged Protein PURIFICATION GEL, while 50 μl of the supernatants remained on ice as the 10% input. After the samples were mixed by inverting at 4 °C for 1 h, the agarose was washed three times with 0.1% TX-100 buffer (50 mM Tris-HCl pH7.5, 150 mM NaCl, 0.1%[v/v] Triton X-100). Bound proteins were eluted with SDS-PAGE sample buffer containing DTT.

### *In vitro* phosphatase assay

Cells grown in 6-well plates were washed twice with PBS and solubilized with 1% CHAPS buffer (50 mM Tris-HCl pH7.5, 150 mM NaCl, 1% [w/v] CHAPS, protease inhibitor cocktail) on ice for 10 min. Insolubilized debris was removed by centrifugation at 12,000×*g* for 2 min at 4 °C and the supernatants were divided into two aliquots. One aliquot was mixed with 30U of calf intestine alkaline phosphatase and the other was mixed with PhosSTOP and incubated for 30 min at 37 °C.

### Statistical analysis

Statistical analyses were performed using data obtained from three or more biologically independent experimental replicates. Student’s *t* test was used for comparisons between two groups and Dunnett’s test was used for multiple comparisons using GraphPad Prism (n.s., not significant; ∗, *p* < 0.05; ∗∗, *p* < 0.01; ∗∗∗, *p* < 0.001).

## Data availability

All data are included in the manuscript.

## Supporting information

This article contains supporting information (9, 20, 23).

## Conflict of interest

The authors declare that they have no conflicts of interest with the contents of this article.

## Supporting information

Supplemental Table S4

Fig 1C-1

Fig 1C-2

Fig 2A

Fig 2B

Fig 2C

Fig 2D

Fig 3B

Fig 3D

Fig 3F

Fig 4A

Fig 5A

Fig 5B

Fig 5C

Fig 5E

Fig 5G

Fig 5I
