# Supplementary figures and images for: TBK1 adaptor AZI2/NAP1 regulates NDP52-driven mitochondrial autophagy
Source: J Biol Chem. 2024 Sep 12;300(10):107775. doi: 10.1016/j.jbc.2024.107775 (PMC11490886; doi:10.1016/j.jbc.2024.107775)

Fig 1C NT

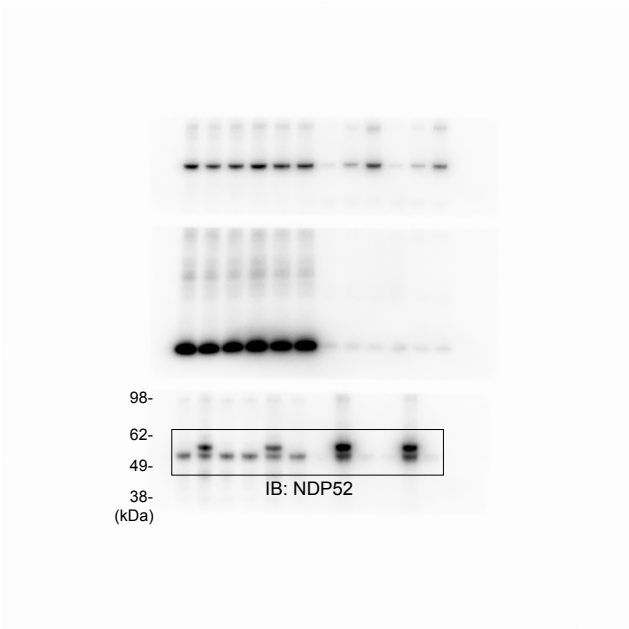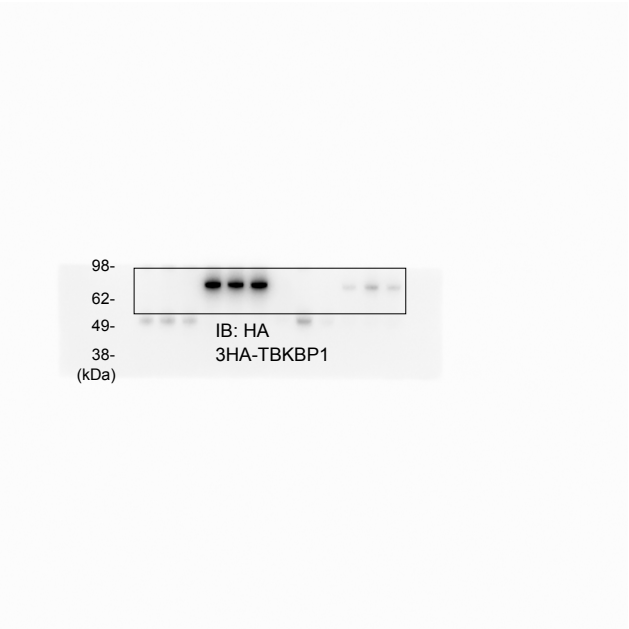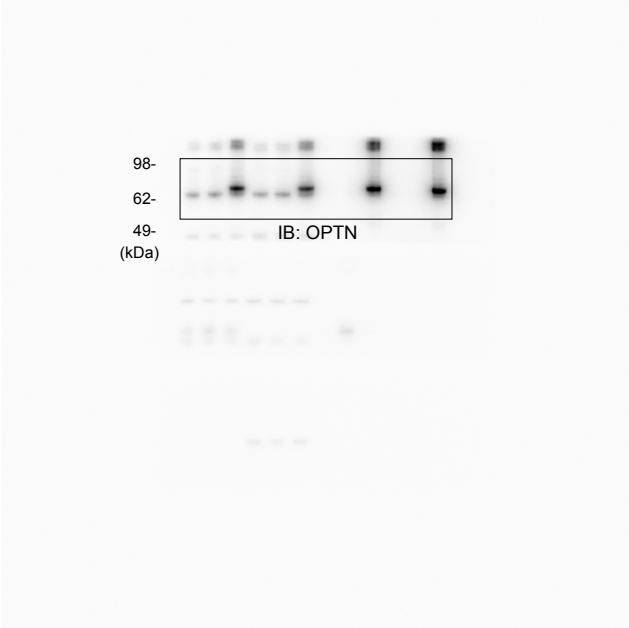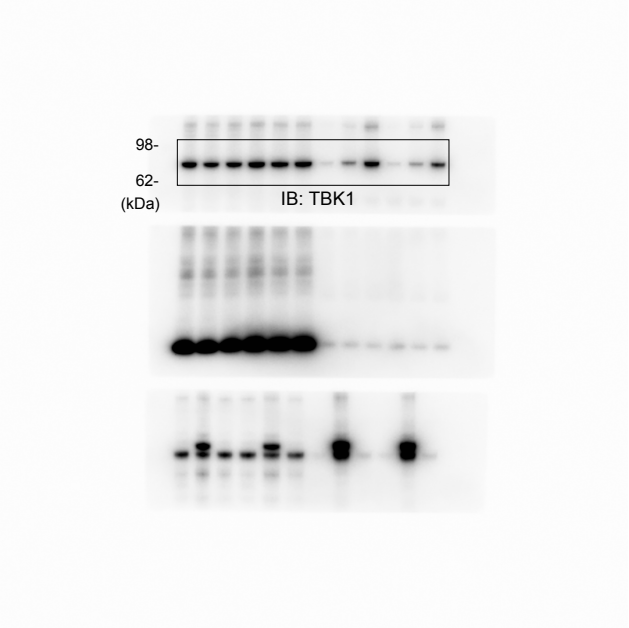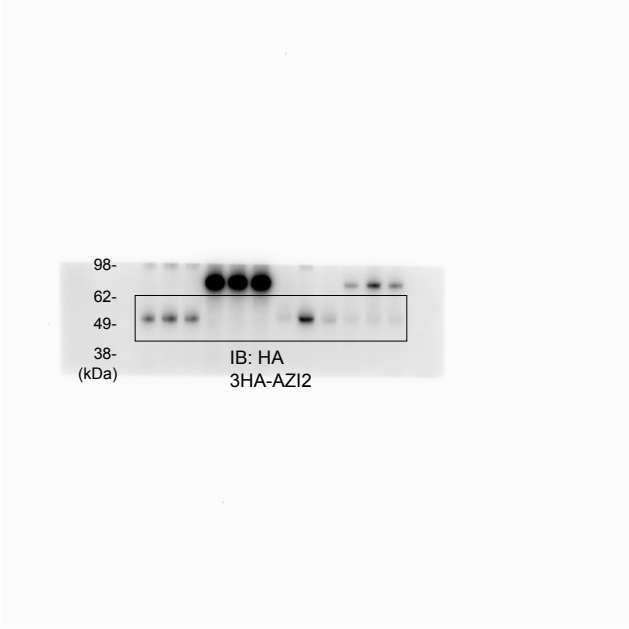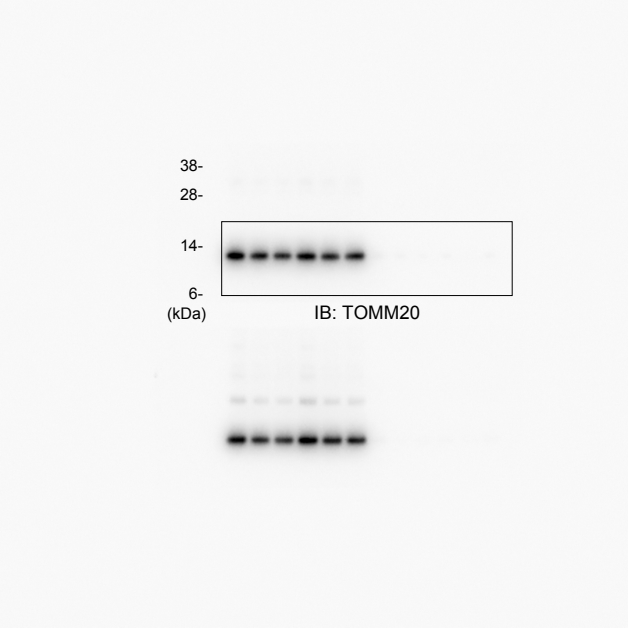

Supplement: Fig 1C-1 [file mmc5.pdf]

Fig 1C val 30min

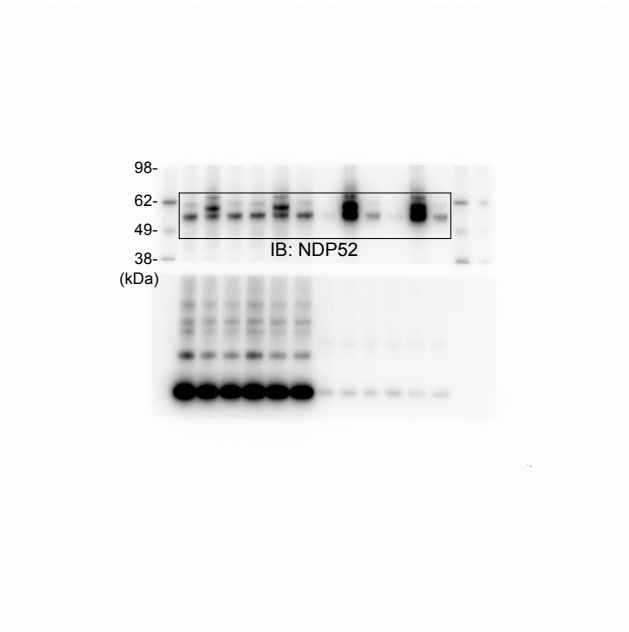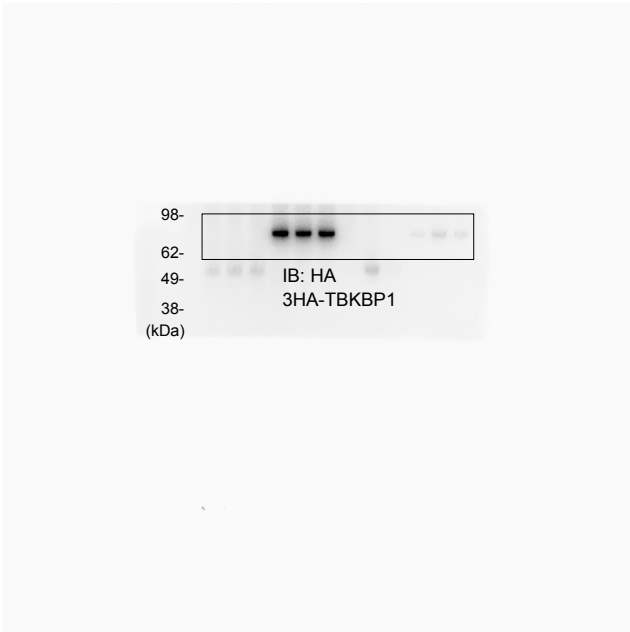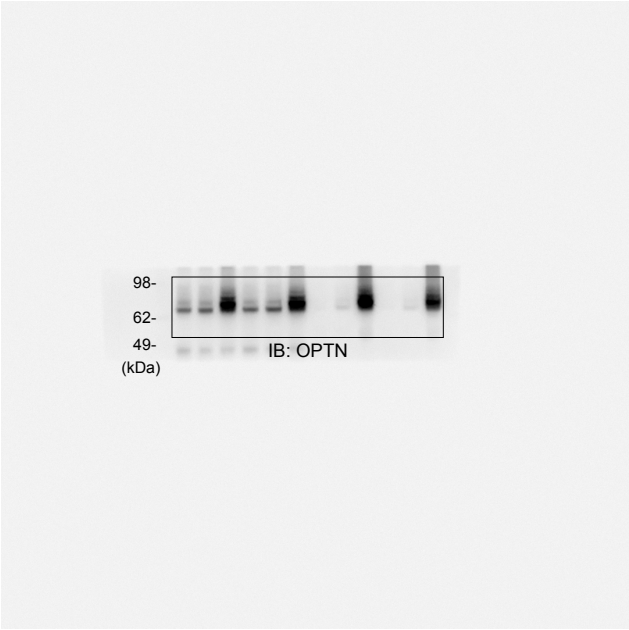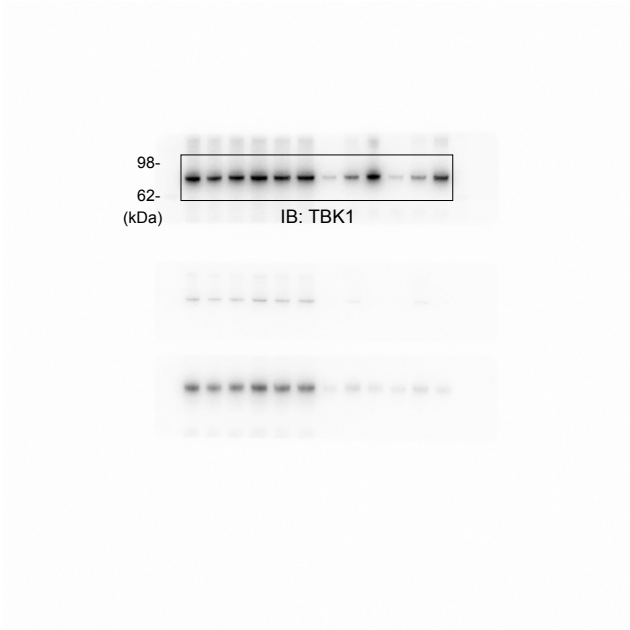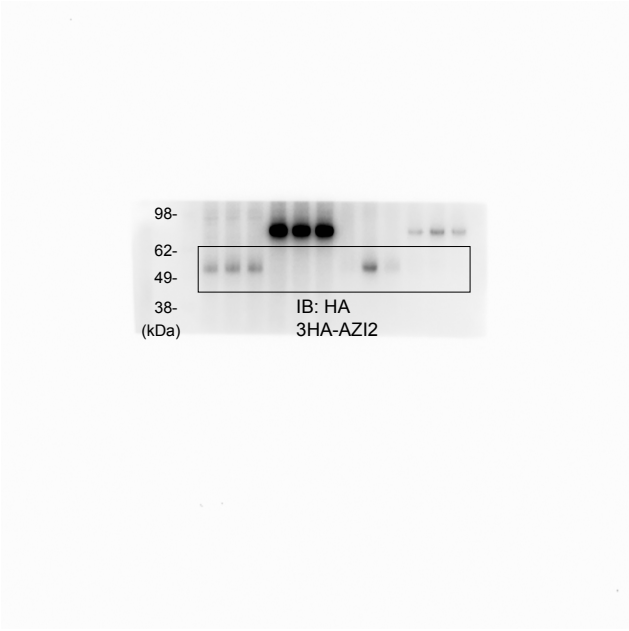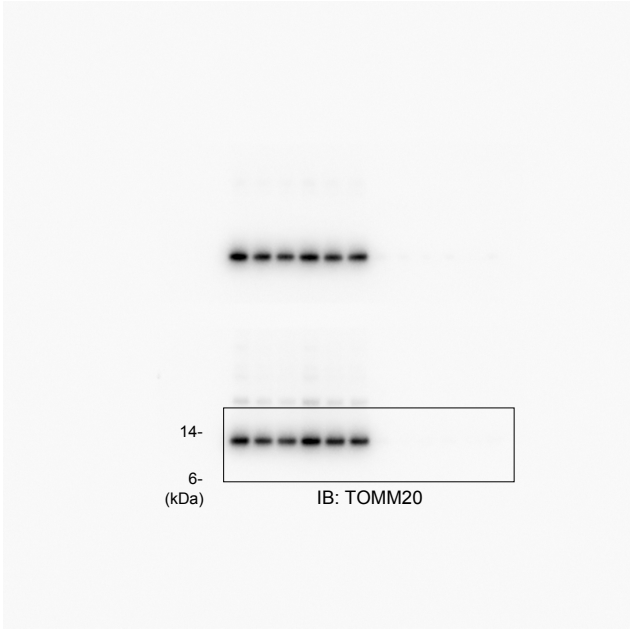

Supplement: Fig 1C-2 [file mmc6.pdf]

Fig 2A

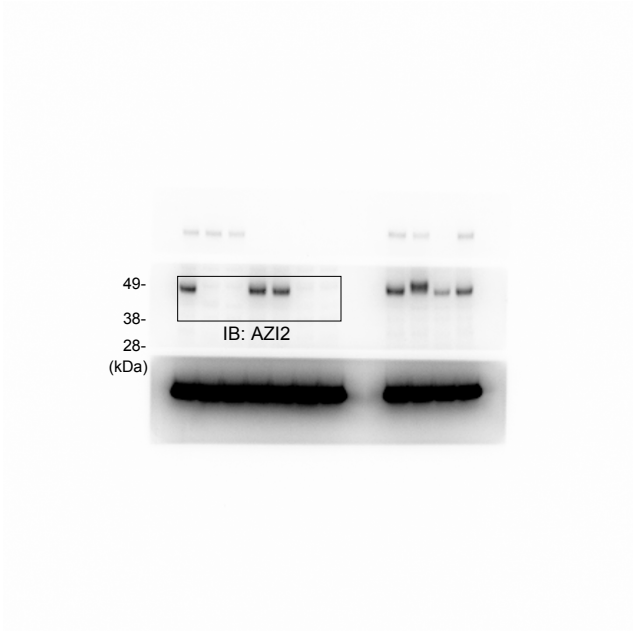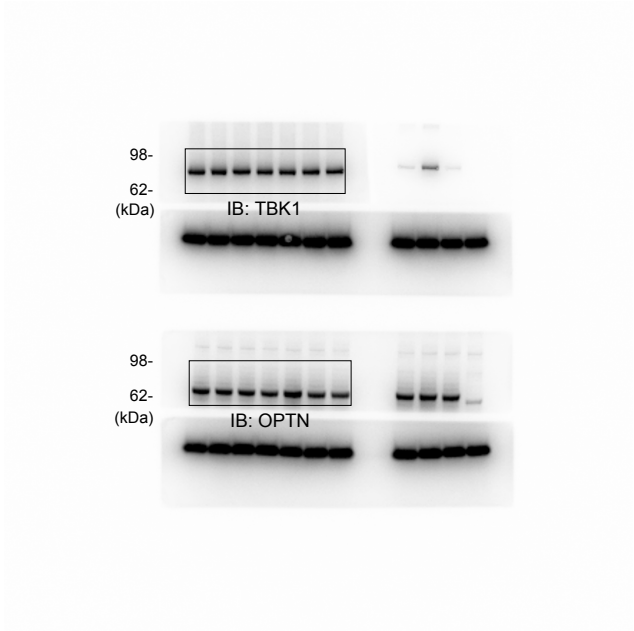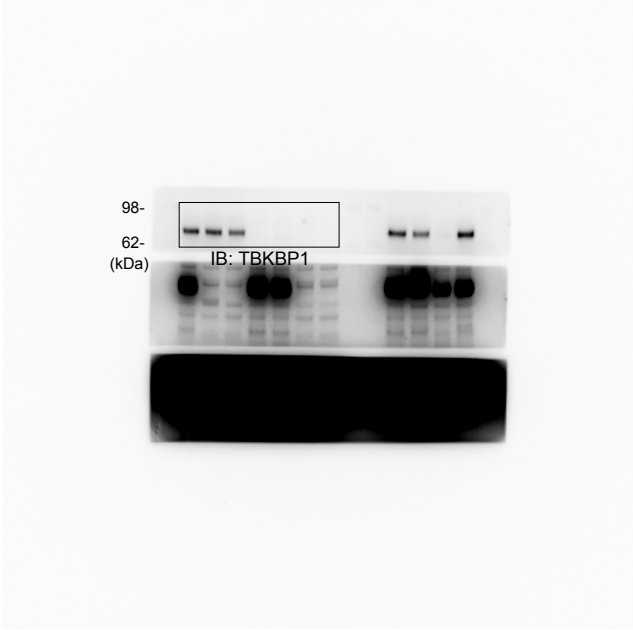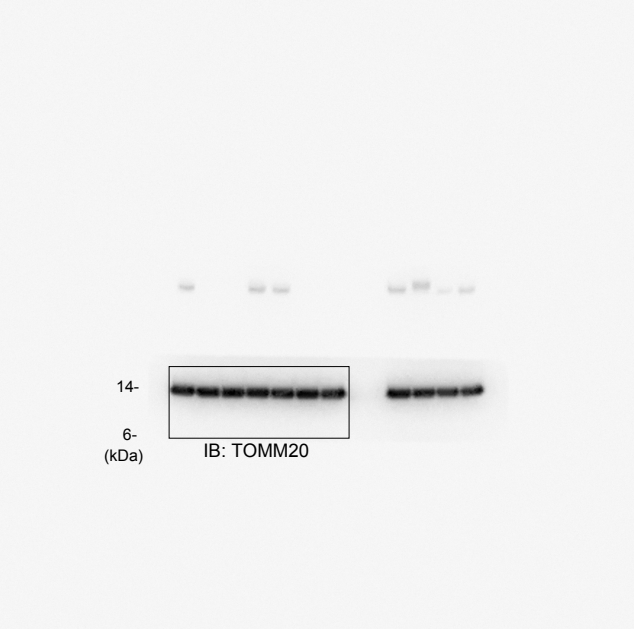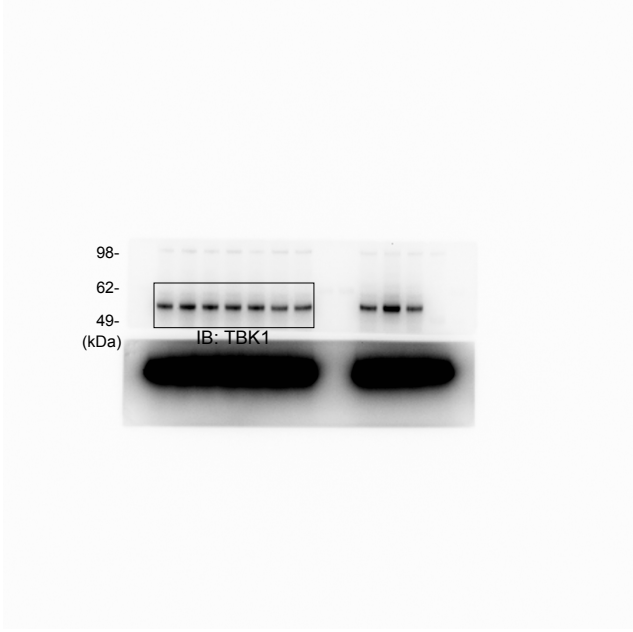

Supplement: Fig 2A [file mmc7.pdf]

Fig 2B

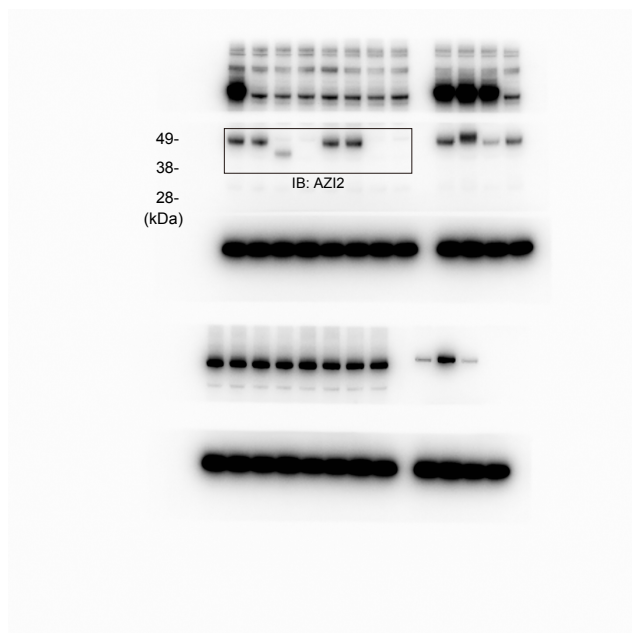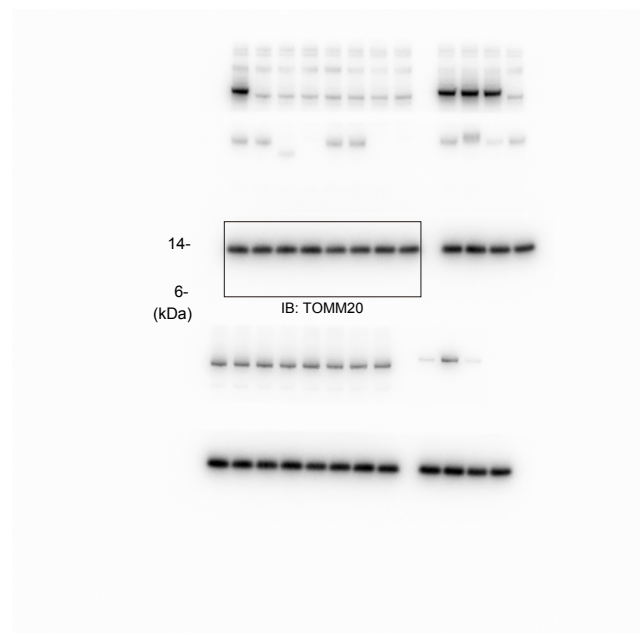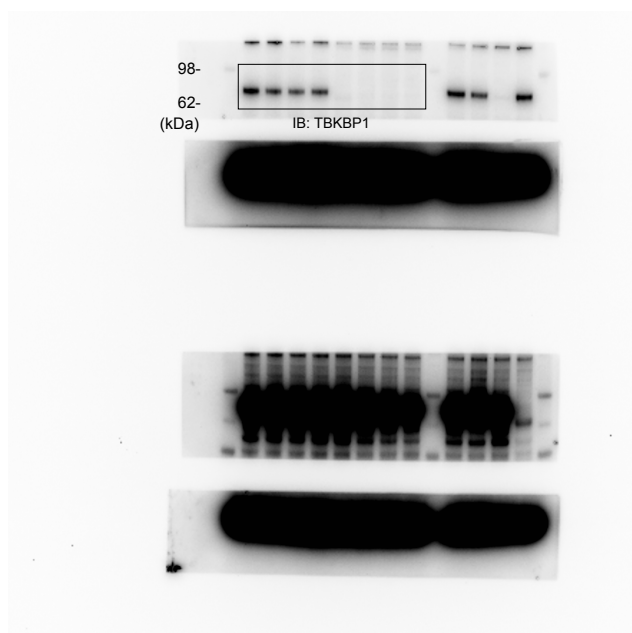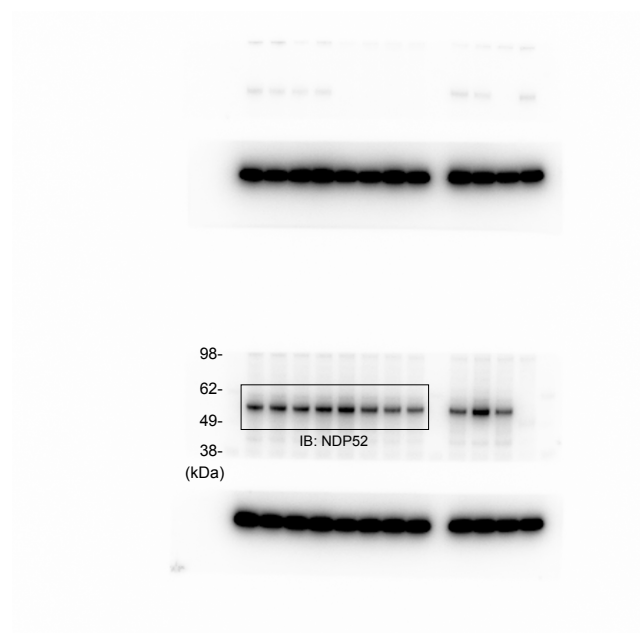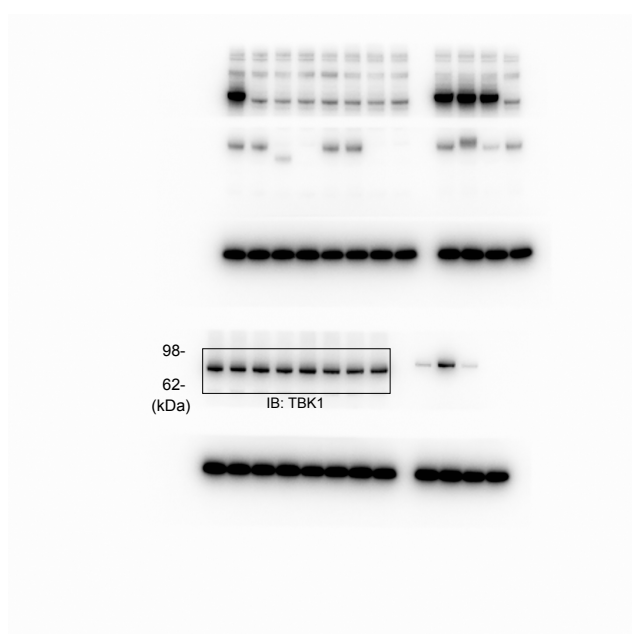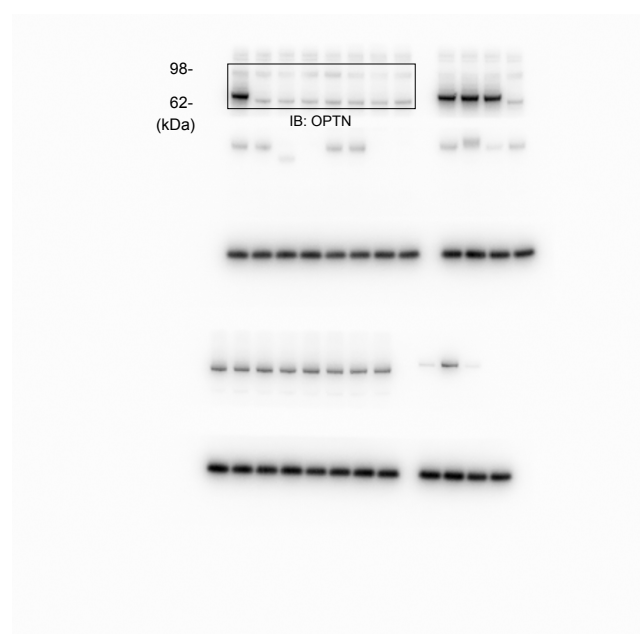

Supplement: Fig 2B [file mmc8.pdf]

Fig 2C

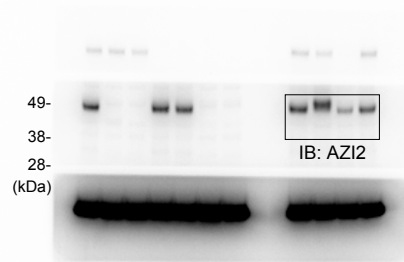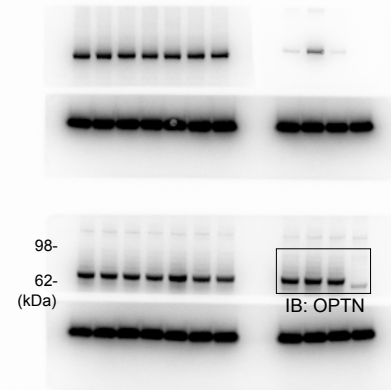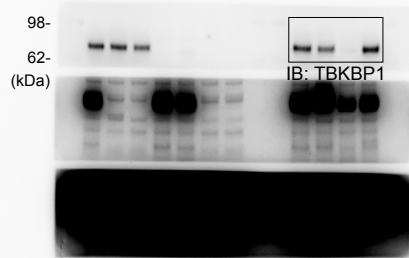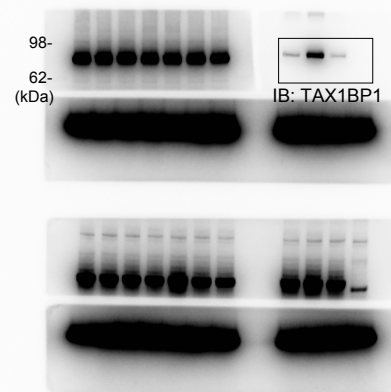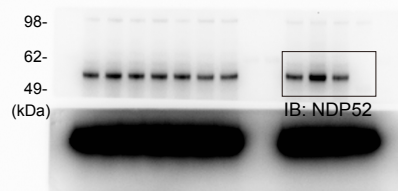

Supplement: Fig 2C [file mmc9.pdf]

Fig 2D

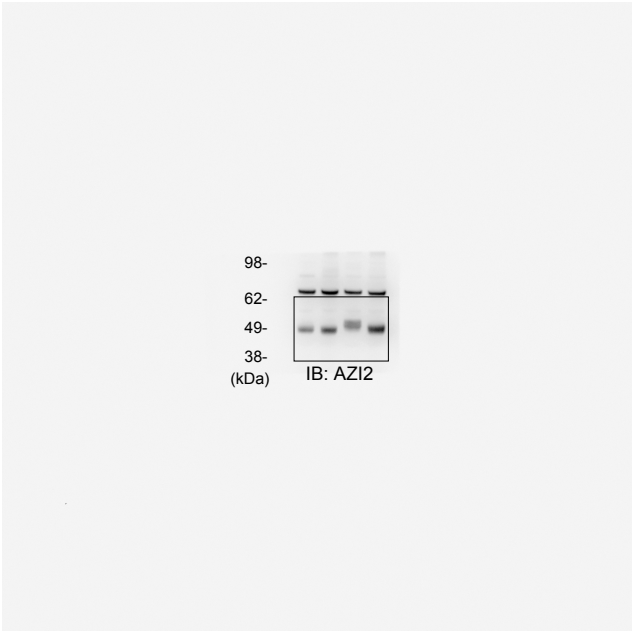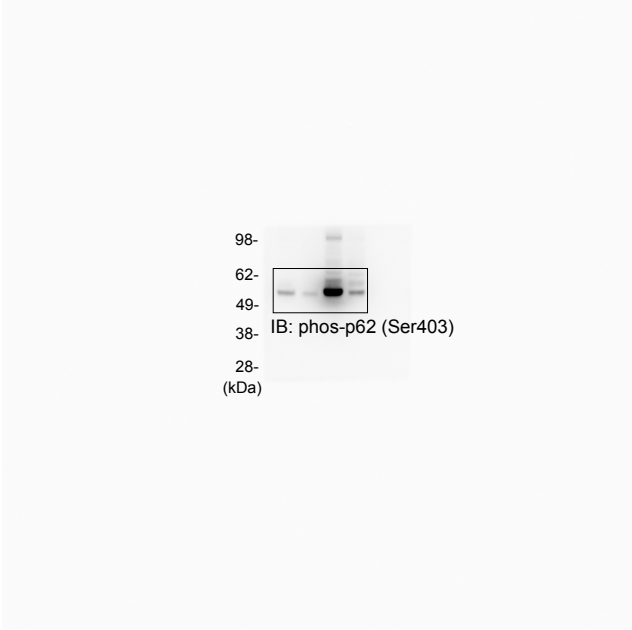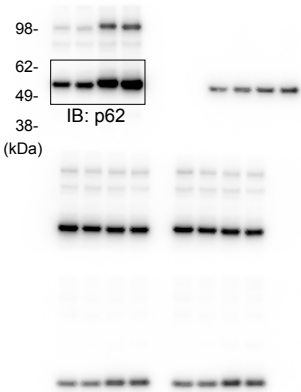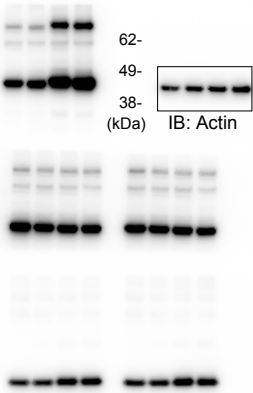

Supplement: Fig 2D [file mmc10.pdf]

Fig 3B

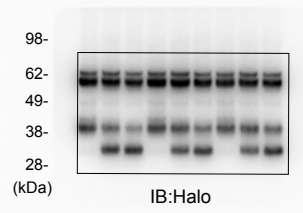

Supplement: Fig 3B [file mmc11.pdf]

Fig 3D

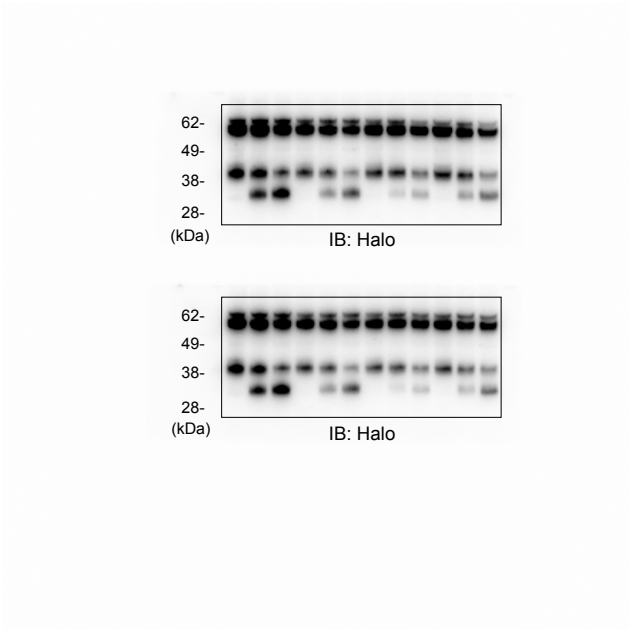

Supplement: Fig 3D [file mmc12.pdf]

Fig 3F

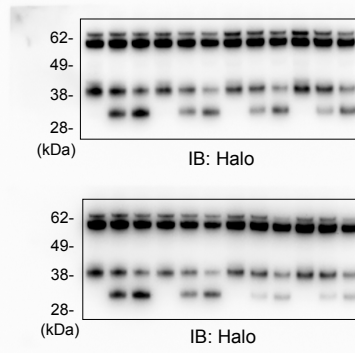

Supplement: Fig 3F [file mmc13.pdf]

Fig 4A

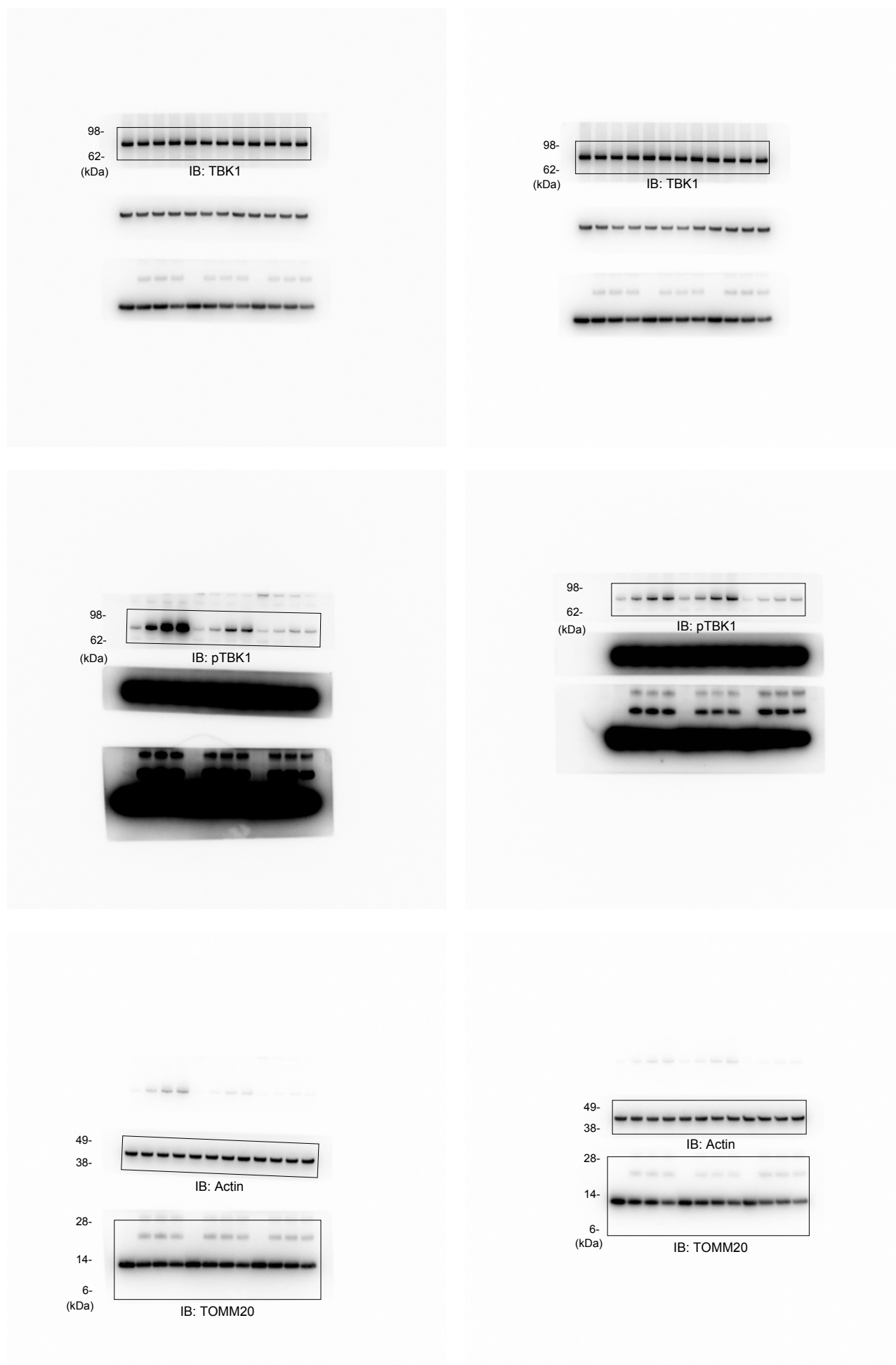

Supplement: Fig 4A [file mmc14.pdf]

Fig 5A

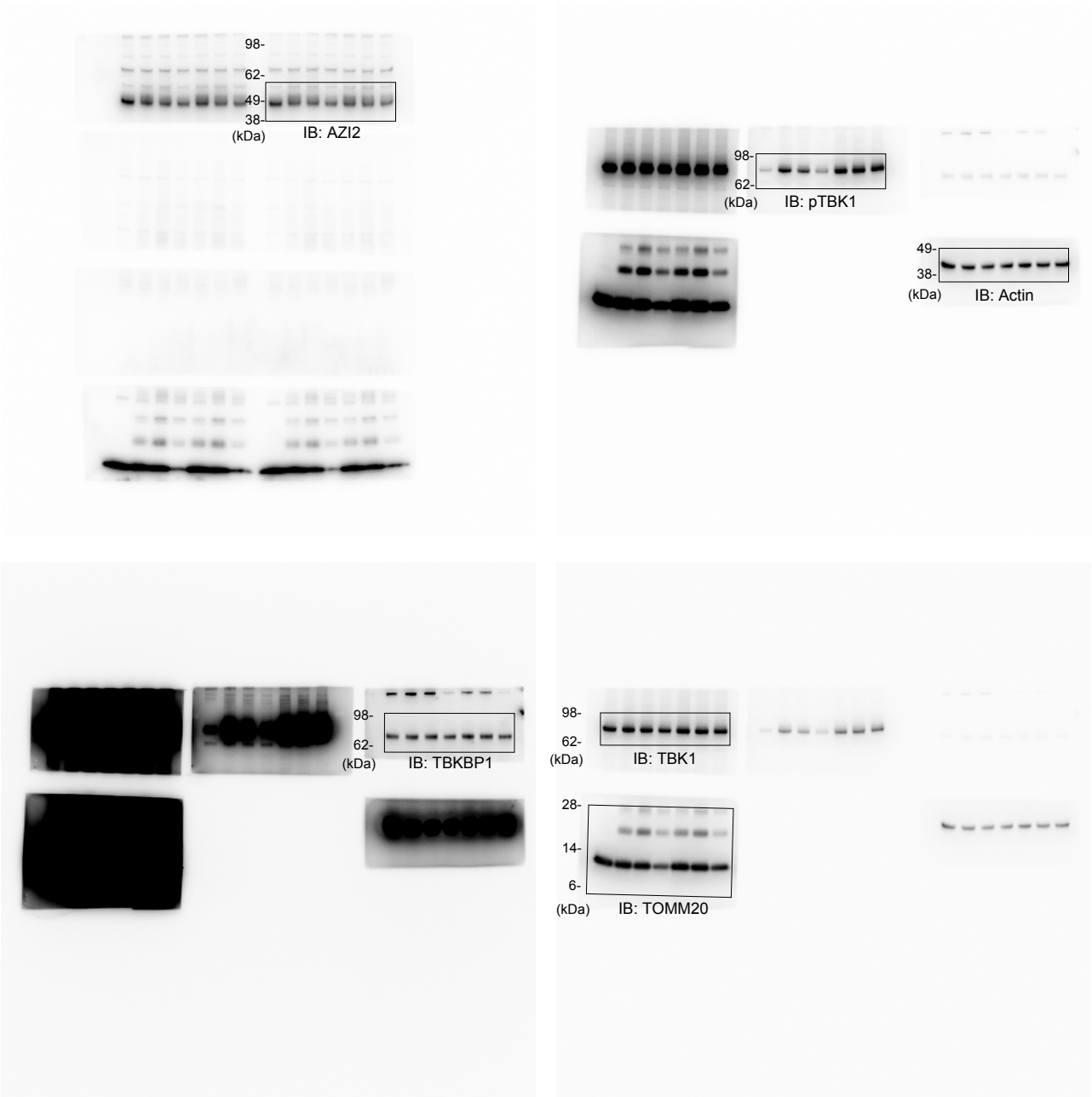

Supplement: Fig 5A [file mmc15.pdf]

Fig 5B

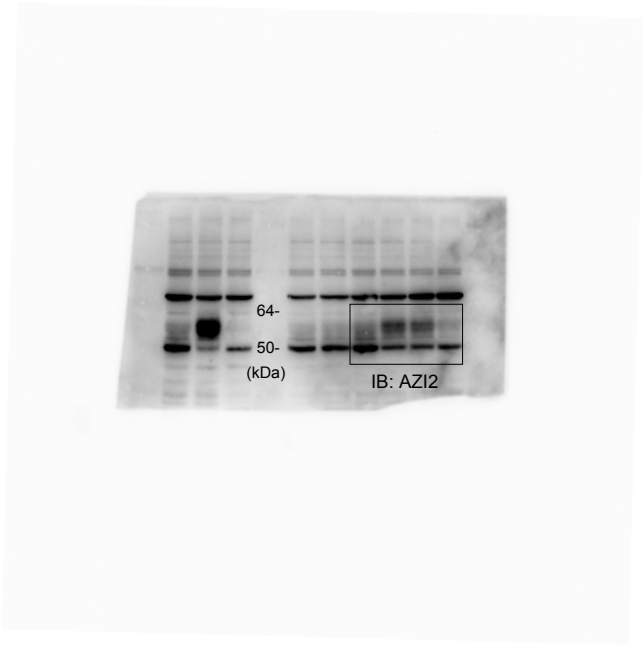

Supplement: Fig 5B [file mmc16.pdf]

Fig 5C

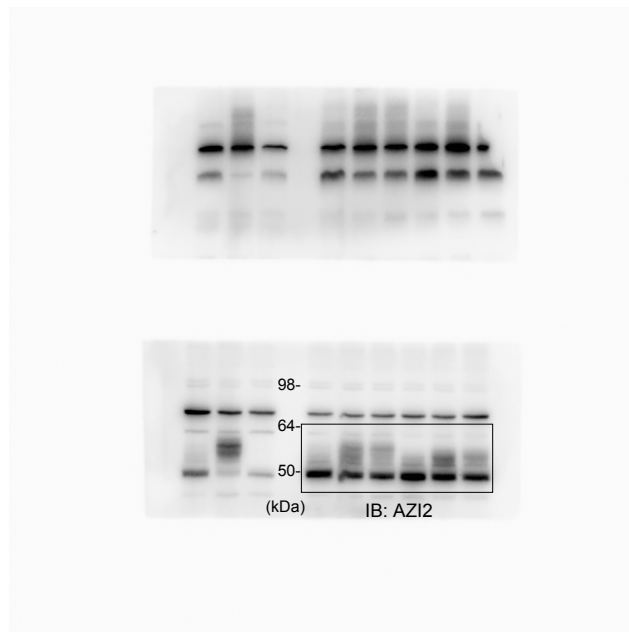

Supplement: Fig 5C [file mmc17.pdf]

Fig 5E

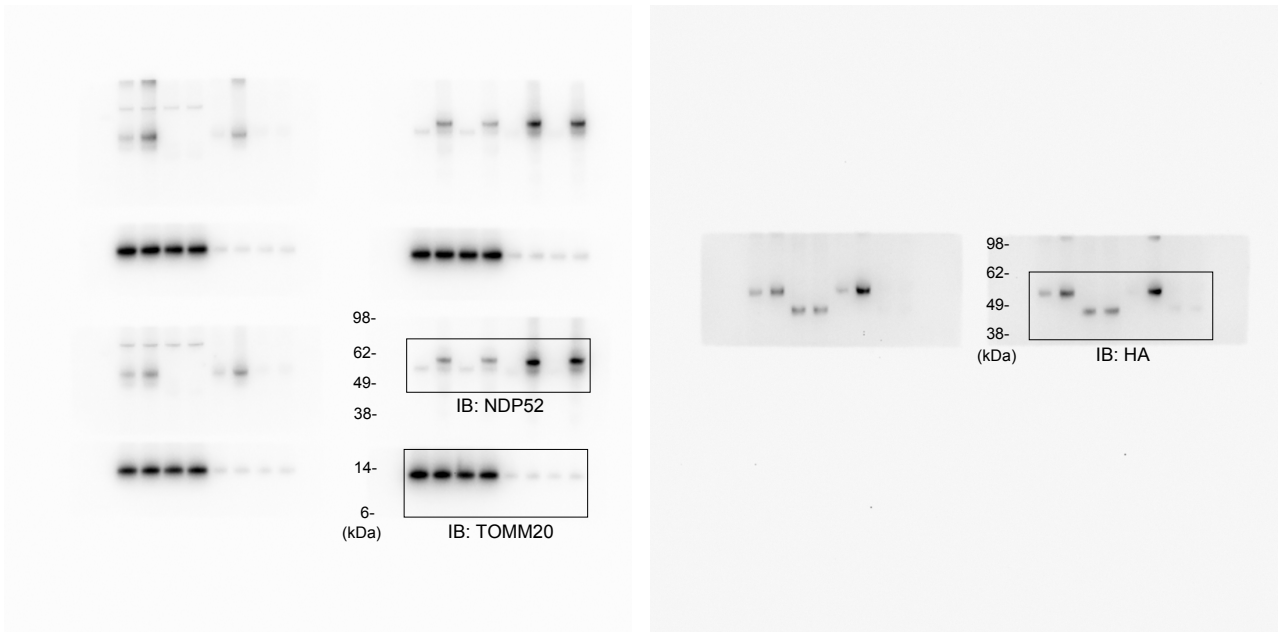

Supplement: Fig 5E [file mmc18.pdf]

Fig 5G

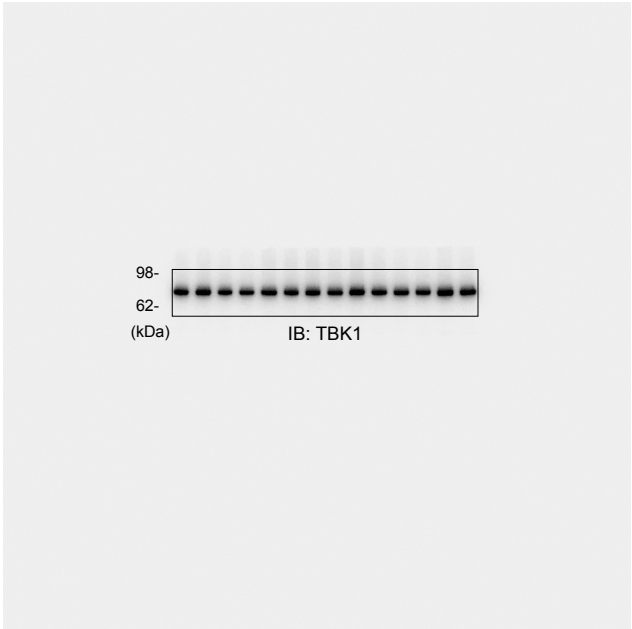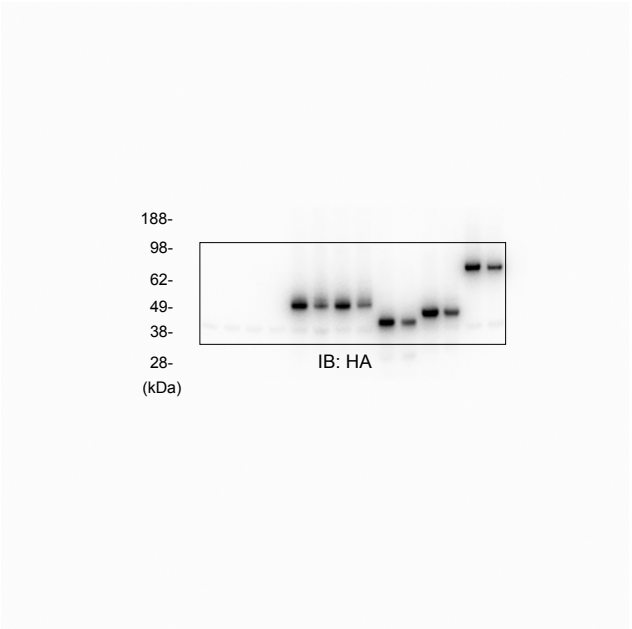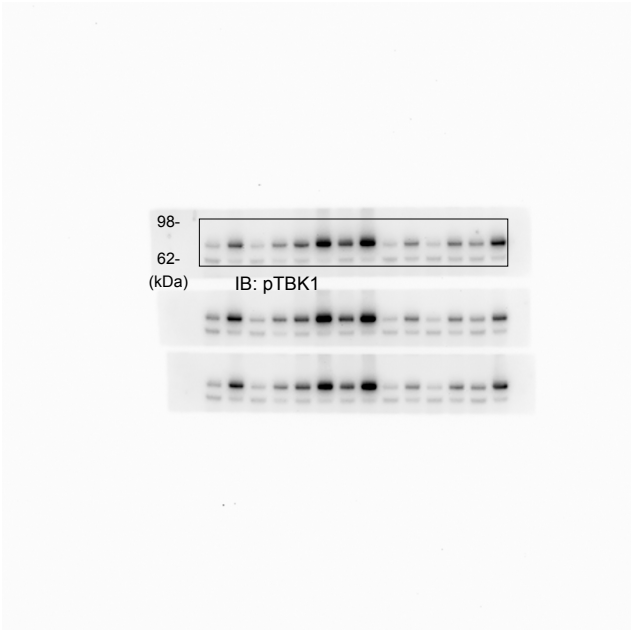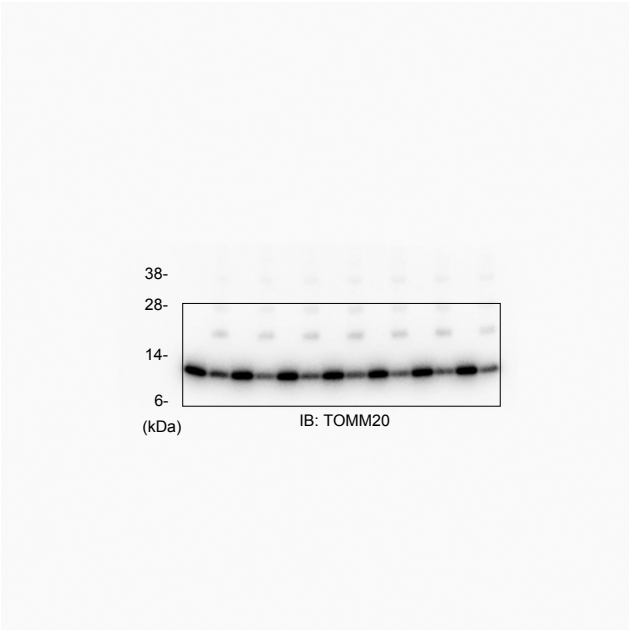

Supplement: Fig 5G [file mmc19.pdf]

Fig 5l

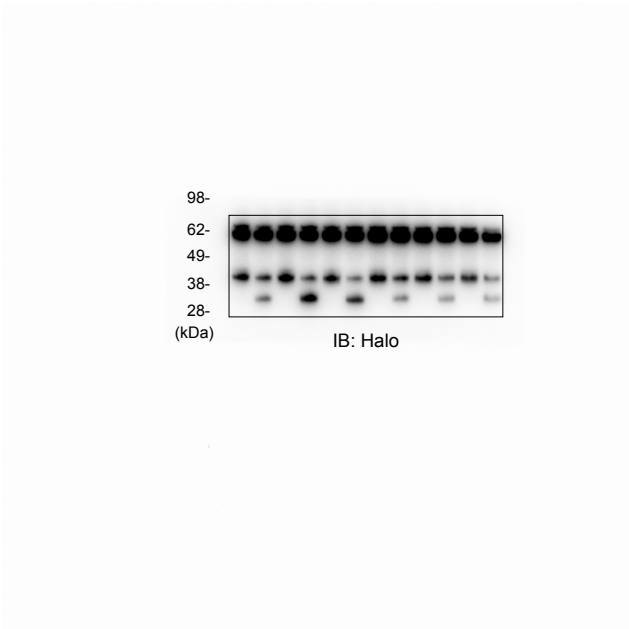

Supplement: Fig 5I [file mmc20.pdf]
